# A text-mining study on emotional cognition, understanding, and preventative behaviors during the COVID-19 pandemic

**DOI:** 10.1186/s12889-023-15180-2

**Published:** 2023-03-03

**Authors:** Eunjung Lim, Jieun Shin, Seyeon Park

**Affiliations:** 1 Department of Nursing, Daejeon Institute of Science and Technology, Daejeon, South Korea; 2 Department of Biomedical Informatics, College of Medicine, Konyang University, Nonsan, South Korea; 3 Department of Nursing, College of Nursing, Chungnam National University Hospital, Daejeon, South Korea

**Keywords:** Covid 19, Emotional cognition, Preventative behaviours, Text-mining

## Abstract

**Background:**

This study aimed to look at emotions perceived about the attributes, prevention, diagnosis, and treatment of infectious diseases related to coronavirus disease (COVID-19) that were widespread across the world and identify their relevance to knowledge about infectious diseases and preventative behaviors.

**Methods:**

Texts to measure emotional cognition were selected through a pre-test, and 282 people were chosen as participants based on the survey conducted for 20 days from August 19 to August 29, 2020, created with Google Forms. IBM SPSS Statistics 25.0 was used for the primary analysis, and the SNA package in R (version 4.0.2) was utilized to conduct the network analysis.

**Results:**

It was found that universal negative emotions such as feeling “anxious” (65.5%), “afraid” (46.1%), and “scared” (32.7%) commonly appeared among most people. Also, they were found to be feeling both positive (“caring” [42.3%] and “strict” [28.2%]) and negative (“frustrating” [39.1%] and “isolated” [31.0%]) emotions about efforts to prevent and curb the spread of COVID-19. In terms of emotional cognition for the diagnosis and treatment of such diseases, “reliable” (43.3%) took the biggest ratio among the replies. The level of understanding about infectious diseases showed differences in emotional cognition, thereby affecting people’s emotions. However, no differences were found in the practice of preventative behaviors.

**Conclusions:**

Emotions associated with cognition in the context of pandemic infectious diseases have been found to be mixed. Furthermore, it can be seen that feelings vary depending on the degree of understanding of the infectious disease.

## Background

The advancement of modern society, globalization, rapid demographic movement, changing human behaviors, and climatic and ecological changes on Earth have led to the development of symptoms and signs of new infectious diseases for which there are no vaccines or remedies, which has led them to spread widely globally [1–3].

Korea has suffered from several new infectious respiratory diseases that spread through droplets and contacts, such as Severe Acute Respiratory Syndrome (SARS) in 2002, H1N1 in 2009, Ebola in 2014, Middle East Respiratory Syndrome-related Coronavirus (MERS-CoV) in 2015, and Coronavirus (COVID-19) in 2019. As the outbreak and spread of the diseases put Koreans in fear and threatened their safety, people began to take more interest in newly-created infectious respiratory diseases. Moreover, with the growing importance of managing and responding to respiratory-related infectious diseases, national disease control efforts entered a new phase [2, 4, 5].

As infectious respiratory diseases become more prevalent, people’s concerns and fears over infection have become a complex issue that affects society as a whole, no longer being a mere medical problem. As COVID-19 continued to spread worldwide, the World Health Organization (WHO) declared it an epidemic on March 11, 2020 in consideration of its seriousness [4]. Hence, the Korea Disease Control and Prevention Agency (KDCA) called for preventative behaviors to be taken at an individual level, such as wearing a mask, washing hands after returning home, refraining from traveling to endemic areas, trying to refrain from coughing outside, and practicing social distancing [6]. However, regardless of national efforts to restructure the national crisis response system, improve public awareness about preventative behaviors by running campaigns in case of outbreaks, and build mutual cooperation among crisis response organizations at home and abroad to take rapid action upon any spread of COVID-19, as well as education and right response, anxiety over COVID-19 still exists [7].

The emotional discomfort that appeared upon the outbreak of COVID-19, such as anxiety, fear, sorrow, and anger varied among individuals. Korea has been identifying the movements of confirmed patients through epidemiological investigations and managing those subject to self-isolation when their movements overlap with confirmed patients. Confirmed and self-isolated people may experience fear of the consequences of COVID-19 infection, and patients in self-isolation may experience boredom, depression, loneliness, and anger [8]. Liu et al. [9] found that group tragedies due to infectious diseases cause many people to suffer from psychological problems, such as depression, anxiety, and stress [10]. However, if an individual lacks emotional cognition, they may have fewer elements to appropriately respond to relevant situations. Therefore, individuals’ emotional cognition can be an important issue in national efforts to manage infectious diseases. [11] Emotional cognition is the ability to accurately realize one’s own emotions and further accurately recognize and empathize with the emotions of others. An individual can control and respond appropriately to their feelings only by clearly recognizing their emotional state. However, if emotional cognition of COVID-19 is unclear, people fail to acknowledge their true feelings, and if they are closed to their own emotions, they will fail to realize their emotional status and have greater difficulties empathizing with others’ feelings. As this can lead to poor adaptation to COVID-19, affecting preventative behaviors, emotional cognition can be a significant element in managing those diseases [11, 12].

When there is a national crisis, such as an outbreak of COVID-19, people realize the severity of the disease through subjective judgment, which leads to behaviors to minimize and evade the impact. This includes the search for information and understanding [13]. Jung, Yoon, and Choi [14] analyzed the prevalence of H1N1 in 2019 and 2010 and response cases involving 1,569 adults aged 18 and above in the United States. The results showed that individuals with good knowledge and interest in the H1N1 virus, who have a robust health-related social network and seek health information from their medical team, have a greater likelihood of engaging in non-pharmaceutical interventions such as social distancing and maintaining personal hygiene than their counterparts. Moreover, Kim et al. [15] analyzed factors that affect the frequency of handwashing for the prevention of new influenza; people with greater anxiety tended to wash their hands more frequently. This implies that, as hand washing depends on self-control, it can be an important behavior in motivating people to prevent infection. In summary, it was found that people who feel greater anxiety, fear, and stress about infectious diseases and people with a better understanding of them have a higher intention to practice preventative behaviors and take a greater interest in them.

Many studies report that taking emotional factors into account was necessary when it came to preventative behaviors for infectious diseases [16–18]. Also, while several studies have been conducted in Korea on the topics of awareness, attitudes, and preventative behaviors for new infectious respiratory diseases [3, 5, 19], no studies have yet attempted to analyze emotional cognition related to anxiety, fear, and stress that people feel about COVID-19. Visualizing the research results by deriving and analyzing texts on emotional cognition using social network analysis helps obtain meaningful results when analyzing social phenomena related to COVID-19. Uncertain and unpredictable situations such as the outbreak of COVID-19 are especially known to have an impact on not only physical but also mental health. [20] Therefore, as the sudden onset of infectious disease can cause anxiety, depression, and stress in people, it is important to provide appropriate guidelines for mental health, taking psychological status as a critical factor.

Hence, this study carried out text-mining, where the team first collected texts (words) exhibiting the emotional cognition of people toward COVID-19 and performed social network analysis using the graph theory to find out their relationship with emotional cognition, understanding, and preventative behaviors.

This research aimed to study emotional cognition that can affect COVID-19 and identify the relevance among words of emotional cognition and the relationship between those words and emotional cognition, understanding, and preventative behaviors related to COVID-19. The detailed objectives are as follows:

1. Identify general characteristics of the subjects.
2. Identify texts on emotional cognition for COVID-19 and find relevance among the texts.
3. Identify correlation among emotional cognition, understanding, and preventative behaviors related to COVID-19.

## Methods

### Design

This study is a descriptive research study designed to look at emotional cognition that can impact the management of COVID-19 and determine its correlation with emotional cognition, understanding, and preventative behaviors.

The scope of the research was divided into three parts following the guidelines for COVID-19 announced by the KDCA in 2020 (cognition and characteristics of COVID-19; prevention and spread control of COVID-19; and diagnosis and treatment of COVID-19). Texts related to the three parts were selected, and closeness centrality was identified among the words by analyzing their usage frequency through the survey.

This study focused on emotional cognition related to Severe Acute Respiratory Syndrome (SARS) in 2002, H1N1 in 2009, Middle East Respiratory Syndrome-related Coronavirus (MERS-CoV) in 2015, and Coronavirus diseases (COVID-19) in 2019, which are the representative coronavirus cases in Korea. Moreover, 20 most commonly used texts in thesis papers, newspapers, online news articles, and social media were extracted for the survey, and through the pre-survey of 30 adults aged 19 or older, 10 texts were finally selected. Five texts expressing feelings about COVID-19 among the selected texts were collected for each part—15 in total.

### Participants

This study included adults aged 19 or older, and the age distribution according to gender was similar. A total of 284 people were included in the final analysis, excluding five duplicate respondents.

### Tools

To measure the “emotional cognition” regarding understanding knowledge and preventative behaviors that can impact the management of infectious diseases, the subjects’ feelings about the attributes, prevention and spread control (quarantine, self-quarantine, preventative efforts, etc.), and diagnosis and treatment of COVID-19 were studied. Considering that the response rate could be low when the survey takes an open format as perceived emotions vary from individual to individual, a pre-test was performed on major emotions. Open questions were given to 30 subjects, and 10 texts (about emotional cognition) that they most frequently replied to in areas of the diseases’ characteristics, prevention, spread control, diagnosis, and treatment were utilized for this study.

### Data collection

The data were collected over a 20-day period through Google Forms from August 19 to August 29, 2020. The researchers explained the objective of the study to the participants and clarified that the results will not be used for other purposes and the provided information will be kept confidential. Furthermore, it was noted that there would be no disadvantage even if the participant withdrew from the study, and the decision to take part in the study was made voluntarily. Participants who indicated an intention to take part in the study filled out an online consent form and a questionnaire.

### Data analysis

IBM SPSS Statistics 25.0 was used for the frequency analysis of the participants’ characteristics as well as the characteristics of their emotions regarding prevention and spread control (such as quarantine, self-quarantine, preventative efforts), and diagnosis and treatment of COVID-19. To examine the orders among the emotions felt about the characteristics, prevention, spread control diagnosis, and treatment of COVID-19, the SNA package of R (version 4.0.2) was used to perform the network analysis [21]. The text network analysis was then carried out, a method that allows researchers to identify the relationships between texts through lines linking nodes that match certain keywords. Following this, a network graph was drawn based on the degree of centrality, which is based on local centrality among centrality indicators.

## Results

### Characteristics of the participants

The majority of the participants were women (58.5%). In terms of age, 27.1%, 20.1%, 19%, and 6.7% were in their 30 s and 40 s, 20 s, 50 s, and 60 or older respectively.

Regarding smoking status, which is a health behavior, "never smoked" represented the largest proportion (69.7%), which was followed by “currently smoking” (13.7%) and “smoked before but have quit” (16.5%). Regarding drinking status, “never drink” and “once a month” each accounted for 28.5%, followed by “once a week” (21.5%), “twice a week” (14.1%), and “three times or more a week” (7.4%). Furthermore, those who exercised three or more times per week accounted for 43.7%, and those who slept 6–7 h took the highest proportion of 37.7% (Table 1).

**Table 1:** Characteristics of the subjects

			(*n* = 284)
Characteristics	Categories	Frequency	Percentage
Gender	Male	118	41.5
	Female	166	58.5
Age	20–29	57	20.1
	30–39	77	27.1
	40–49	77	27.1
	50–59	54	19.0
	60 and above	19	6.7
Smoking Status	Smoked before but have quit	47	16.5
	Currently smoking	39	13.7
	Never smoked	198	69.7
Drinking Status	Never drink	81	28.5
	Once a month	81	28.5
	Once a week	61	21.5
	Twice a week	40	14.1
	Three times or more a week	21	7.4
Exercise	I don’t exercise	160	56.3
	I exercise	124	43.7
Sleep	5 h or below	28	9.9
	5–6 h	79	27.8
	6–7 h	107	37.7
	7–8 h	65	22.9
	More than 8 h	5	1.8

### Understanding of COVID-19

An average of 18.7 (SD = 2.9) out of 25 questions on understanding infectious diseases were found to have been correctly answered. The items that showed the highest percentage of correct answers were “Washing hands with soap and water for 30 s can help prevent the spread of COVID-19” and “I cover my mouth with a sleeve if I don’t have a tissue or handkerchief.” Meanwhile, the items with 50% or more incorrect answers were “The incubation period for COVID-19 is 1–3 days,” “Plants are the main carriers for the transmission of COVID-19,” “You must wear a mask once you start taking medications, not beforehand when you have respiratory symptoms,” and “Certified health masks (such as KF94, KF80) can be used for more than 1 day,” implying that the participants’ understanding of the treatment or medical knowledge of COVID-19 was low (Table 2).

**Table 2:** Understanding of COVID-19 (*n* = 284)

Questions	Wrong	Don’t know	Correct	Rank
SARS, MERS, and COVID-19 are severe acute respiratory-related infectious diseases	3.2	7.4	89.4	7
Major symptoms of COVID-19 are fever, cough, sore throat, and difficulty in breathing	2.1	4.2	93.7	6
Blood and body fluid of all patients with COVID-19 should be treated as a potential source of infection	14.4	13.0	72.5	20
People with underlying diseases (diabetes, chronic lung diseases, cancer, heart failure, etc.) are more likely to be infected	15.1	7.4	77.5	18
The incubation period for COVID-19 is 1–3 days	51.8	20.4	27.8	23
COVID-19 spread through close contact with the infected	13.7	2.1	84.2	12
Plants are the main carriers of the transmission of COVID-19	72.9	21.1	6.0	25
Washing hands with soap and water for 30 s can help prevent the spread of COVID-19	2.1	1.1	96.8	1
Hospital treatment of COVID-19 is carried out in negative pressure isolation rooms or medical institutes with isolated facilities	1.4	2.8	95.8	3
You must wear a mask once you start taking medications, not beforehand when you have respiratory symptoms	76.8	11.6	11.6	24
Infectious diseases spread through saliva, runny nose, sputum, etc	3.9	2.1	94.0	5
You only have to cover your mouth when coughing*	10.2	9.5	80.3	16
You can cover the mouth with your hand when coughing*	7.7	9.9	82.4	15
I cover my mouth with a sleeve if I don’t have a tissue or handkerchief	2.5	0.7	96.8	1
It is okay to only cover the mouth when wearing a mask*	3.2	9.9	87.0	10
You can touch the outer surface of the mask after wearing it*	5.3	11.6	83.1	13
Genera cotton masks can block more than 80% of the flu virus*	24.3	20.1	55.6	21
Certified health masks (KF94, KF80, etc.) can be used for more than 1 day*	40.5	12.0	47.5	22
If the inner surface of certified health masks (KF94, KF80, etc.) are contaminated, you can use them again after washing*	5.6	11.6	82.7	14
If your hands are clean after coughing, you don’t need to wash your hands*	2.8	12.0	85.2	11
You have to wash your hands with soap in running water for 30 s after you cough	4.6	1.1	94.4	4
I spit out the sputum in the trash can as soon as I feel it in my throat*	7.7	12.3	79.9	17
If I have to spit out sputum, I use a tissue	5.3	5.6	89.1	8
I know 6 steps to wash my hands properly	10.2	16.9	72.9	19
It is most effective to use soap and water when washing hands	7.7	4.2	88.0	9

### Management and practice of preventative behaviors for COVID-19

The top three items for the management and practice of preventative behaviors for COVID-19 were “I turn my face away from other people when I cough” (3.71 ± 0.49), which was the highest, followed by “I avoid having close contact with people who have a fever or respiratory symptoms” (3.70 ± 0.48), and “I always wash my hands after returning home or working out to prevent infection” (3.69 ± 0.52). Meanwhile, items that the subjects least practiced were “I sleep for 7 or more hours on mean to maintain health” (2.77 ± 0.79) and “I work out on a regular basis for health, such as to boost immunity” (2.81 ± 0.80) (Table 3).

**Table 3:** Management and practice of preventative behaviors for COVID-19 (*n* = 284)

Questions	Mean ± SD	Rank
I refrain from visiting crowded places	3.40 ± 0.58	7
I avoid having close contact with people who have a fever or respiratory symptoms	3.70 ± 0.48	2
I immediately find a medical facility if I have respiratory symptoms such as fever or breathing difficulties	3.46 ± 0.63	6
I don’t smoke or drink	3.05 ± 0.96	11
I ventilate the room often to keep fresh air inside	3.46 ± 0.58	5
I read promotional materials about infectious diseases frequently	2.98 ± 0.72	12
I work out on a regular basis for health, such as to boost immunity	2.81 ± 0.80	13
I sleep for 7 or more hours on average to maintain health	2.77 ± 0.79	14
I cover my mouth and nose with a tissue when I cough or sneeze	3.11 ± 0.70	10
I wear a mask when I have respiratory symptoms	3.63 ± 0.54	4
I turn my face away from other people when I cough	3.71 ± 0.49	1
When I buy a mask, I check whether it is effective in preventing infectious respiratory diseases (KF94, KF80)	3.36 ± 0.74	9
I always wash my hands after returning home or working out to prevent infection	3.69 ± 0.52	3
I wash my hands with soap in running water for 30 s after coughing	3.40 ± 0.69	8

In terms of the differences in practice depending on the level of understanding of COVID-19, no statistically significant difference was found, as the group with poor understanding recorded (3.31 ± 0.37) whereas the mean of the group with good understanding was 3.34 ± 0.32(t = 0.636, *p* = 0.525).

### Characteristics of emotional cognition related to COVID-19

#### Emotional cognition over the attributes of COVID-19

For the attributes of COVID-19, the participants perceived a mean of 2.8 emotions. “Anxious” accounted for 65.5%, which was followed by “Fearful” (46.1%), “Scared” (32.7%), “Threatening” (25.4%), “Horrific” (24.6%), “Frustrating” (21.5%), “Depressed” (21.1%), “Perplexed” (16.5%), “Painful” (15.1%), and “Critical” (11.6%) (Table 4).

**Table 4:** Distribution of emotional cognition about the attributes of COVID-19 (*n* = 284)

Attributes	Total	Group with poor understanding	Group with good understanding
Painful	43(15.1)	28(16.9)	15(12.7)
Horrific	70(24.6)	50(30.1)	20(16.9)
Perplexed	47(16.5)	28(16.9)	19(16.1)
Fearful	131(46.1)	82(49.4)	49(41.5)
Frustrating	61(21.5)	29(17.5)	32(27.1)
Scared	93(32.7)	57(34.3)	36(30.5)
Anxious	186(65.5)	101(60.8)	85(72)
Depressed	60(21.1)	30(18.1)	30(25.4)
Threatening	72(25.4)	44(26.5)	28(23.7)
Critical	33(11.6)	21(12.7)	12(10.2)

**Fig. 1:**
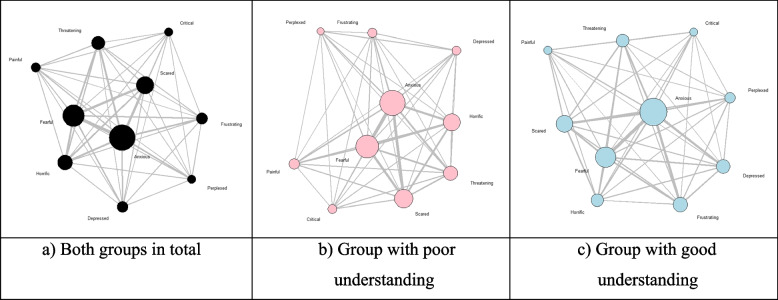
Network graph of emotional cognition over the attributes of “COVID-19”. (Node size: Bigger with a greater degree, Edge thickness: Thicker with a higher frequency of co-occurrence)

Moreover, as shown in Fig. 1, “Anxious,” “Fearful,” and “Scared” appeared to have a high centrality, which is an indicator of local centrality, recording high relevance as well.

Meanwhile, both groups with a poor and good understanding of COVID-19 replied that they felt “Anxious,” “Fearful,” and “Scared” the most. However, there were differences in the lower ranks of emotional cognition over the attributes of COVID-19. For example, while the group with good understanding scored 7%p–12%p higher in universal negative emotions such as “Anxious,” “Frustrating,” and “Depressed,” the group with poor understanding scored approximately 8%p–14%p higher in dramatically negative emotions such as “Fearful” and “Horrific.”

#### Emotional cognition over the prevention and spread control of COVID-19

**Fig. 2:**
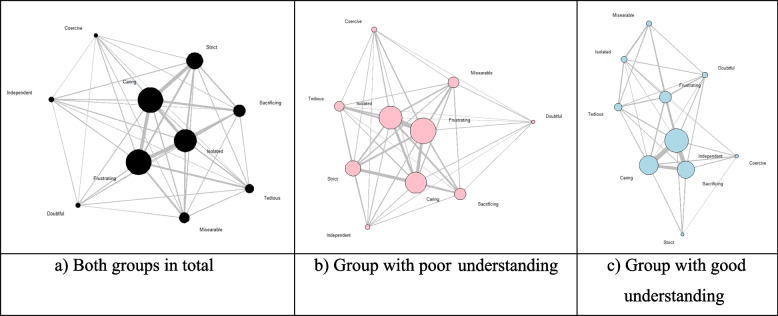
Network graph of emotional cognition over the prevention and spread control of “COVID-19”. (Node size: Bigger with a greater degree, Edge thickness: Thicker with a higher frequency of co-occurrence)

In this category, the participants were found to feel two emotions on average. “Caring” accounted for 42.3%, followed by “Frustrating” (39.1%), “Isolated” (31.0%), “Strict” (28.2%), “Sacrificing” (20.8%), “Miserable” (14.1%), “Tedious” (10.2%), “Doubtful” (7.7%), “Independent” (7.7%), and “Coercive” (5.3%) (Table 5). Additionally, as indicated in Fig. 2, emotions “Caring,” “Strict,” “Isolated,” and “Frustrating” appeared to have a high degree of centrality that indicates local centrality as well as high relevance. Meanwhile, while both groups with a poor and good understanding of COVID-19 felt positive and negative emotional cognitions of “Caring” and “Frustrating” at a high level, the group with a poor understanding recorded about 3%p–5%p higher in negative emotional cognition such as “Isolated,” “Frustrating,” and “Miserable” compared to its counterpart, and the group with a good understanding scored 51.7% in positive emotional cognition such as “Caring,” which was 16%p higher than its poor counterpart. In brief, the findings imply that participants with a good understanding of COVID-19 have more positive emotional cognition. Also, as indicated in Fig. 2, the group with poor understanding recorded a high degree of centrality and relevance in negative emotional cognition whereas the group with good understanding recorded a high degree of centrality in positive emotional cognition.

**Table 5:** Distribution of emotional cognition about the prevention and spread control of COVID-19 (*n* = 284)

Attributes	Total	Group with poor understanding	Group with good understanding
Coercive	15(5.3)	12(7.2)	3(2.5)
Isolated	88(31)	55(33.1)	33(28)
Frustrating	111(39.1)	67(40.4)	44(37.3)
Independent	22(7.7)	13(7.8)	9(7.6)
Tedious	29(10.2)	18(10.8)	11(9.3)
Caring	120(42.3)	59(35.5)	61(51.7)
Doubtful	22(7.7)	10(6)	12(10.2)
Miserable	40(14.1)	25(15.1)	15(12.7)
Strict	80(28.2)	44(26.5)	36(30.5)
Sacrificing	59(20.8)	34(20.5)	25(21.2)

#### Emotional cognition over the diagnosis and treatment of COVID-19

For the diagnosis and treatment of “COVID-19,” the participants perceived an average of 1.8 emotions, where “Reliable” accounted for 43.3%, followed by “Relieving” (32.7%), “Responsible” (29.9%), “Complicated” (16.9%), “Hurtful” (16.2%), “Doubtful” (13%), “Suspicious” (11.3%), “Perilous” (8.8%), “Unruffled” (6.7%), and “Confident” (4.9%) (Table 6).

**Table 6:** Distribution of emotional cognition about the diagnosis and treatment of COVID-19 (*n* = 284)

Attributes	Total	Group with poor understanding	Group with good understanding
Unruffled	19(6.7)	12(7.2)	7(5.9)
Suspicious	32(11.3)	18(10.8)	14(11.9)
Complicated	48(16.9)	25(15.1)	23(19.5)
Reliable	123(43.3)	70(42.2)	53(44.9)
Hurtful	46(16.2)	32(19.3)	14(11.9)
Relieving	93(32.7)	52(31.3)	41(34.7)
Perilous	25(8.8)	14(8.4)	11(9.3)
Doubtful	37(13)	23(13.9)	14(11.9)
Confident	14(4.9)	9(5.4)	5(4.2)
Responsible	85(29.9)	49(29.5)	36(30.5)

**Fig. 3:**
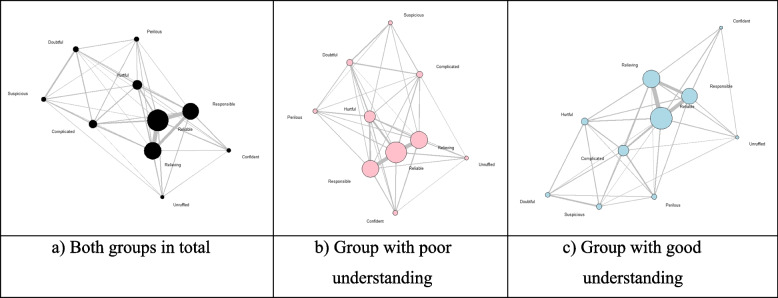
Network graph of emotional cognition over the diagnosis and treatment of “COVID-19”. (Node size: Bigger with a greater degree, Edge thickness: Thicker with a higher frequency of co-occurrence)

As indicated in Fig. 3, positive emotional cognition such as “Reliable,” “Relieving,” and “Responsible” appeared to have a high degree of centrality that indicates local centrality in both groups, which also had a high correlation as shown in the thickness of the edges connecting the nodes.

The emotional cognition of both groups of good and poor understanding of COVID-19 appeared in the same order with a similar degree of centrality and relevance. This implies that in the area of diagnosis and treatment of COVID-19, the subjects perceive positive emotions as “reliable,” “relieving,” and “responsible” regardless of the level of understanding.

## Discussion

This study used text mining to find out what emotions are perceived and related to prevention behavior for infectious diseases according to the level of understanding of respiratory-related infectious diseases, especially coronaviruses, and to discuss the results.

### Emotions based on understanding of infectious diseases

#### Emotions related to infectious disease characteristics

First, regarding infectious disease characteristics, subjects reported feeling "anxiety” (65.5%), and “fear” (46.1%), and “scared” (32.7%). These three words were closely connected, so they were highly related to each other. These results are in the same context as previous studies that have shown that coronaviruses have caused disease and death worldwide and, relatedly, a lot of social damage and anxiety [22]. In addition, the results for emotion recognition, such as "fearful” (24.6%) and "confused” (16.5%), reflect negative emotions of anxiety, fear, and embarrassment, which align with the results of previous studies, such as that by Aslam et al. on emotions related to news headlines about COVID-19 [23]. Existing research suggests that we experience heightened negative emotions, such as fears of death or infection, anxiety, anger, and mental health problems during pandemics [24]. In China, more than half of the study respondents reported considerable mental health consequences due to this traumatic outbreak [25–27]. Another study from Denmark also reported negative psychological consequences from COVID-19, such as depression [28]. Moreover, a survey carried out by the American Psychiatric Association in the United States indicated that nearly half of its respondents experienced psychological problems, such as anxiety, during COVID-19 [29].

Therefore, it is important to ensure accurate information about infectious diseases; if negative emotions persist, psychological stress and tension can increase, leading to a wide range of mental illnesses, such as depression, anxiety disorder, panic disorder, remorse, post-traumatic stress disorder (PTSD), and delirium, and even suicide [1, 30].

In this study, the group with particularly high understanding more commonly recognized that they felt negative emotions, such as "anxiety, hopelessness, and depression," while the group with a low understanding of infectious diseases was higher. Many studies have found that higher levels of education improve skills, cognition, and coping strategies, which all ultimately improve psychological health [31, 32].

It is important to recognize that the characteristics of infectious diseases impact mental and psychological health [33]. In particular, sharing negative emotions with others can emphasize that we all experience similar emotions, which can help us better understand or resolve the situation, thereby reducing mental consumption [34]. Therefore, this study’s first result about the perceived emotions related to infectious coronavirus diseases offers insights useful for best practices for mental health management in the modern pandemic era.

To date, many studies have been conducted on COVID-19-related depression, anxiety, or mental stress from isolation, but there are no studies analyzing emotional recognition or preventive behaviors in relation to understandings of infectious diseases during the uncertain and tense COVID-19 era. This study is the first to consider this topic in terms of emotional recognition, and its strength is that it uses text mining to analyze the relationship and frequency between emotions.

#### Emotions related to quarantine guidelines

Regarding emotions related to respiratory-related infectious disease prevention, our study revealed interesting results.

First, most subjects reported feeling a mix of positive and negative emotions about infectious disease prevention and quarantine. Negative emotions, such as "frustrated” (39.1%) and "isolated” (31.0%) were strongly related to each other; meanwhile, positive attitudes for prevention and quarantine, such as "considerate” (42.3%) and "thorough” (28.2%) were strongly connected locally. Interestingly, participants with a high understanding of infectious diseases was aware of their positive emotions, such as feeling "considerate” (51.7%), while participants with a low understanding of infectious diseases were more aware of their negative emotions, such as feeling "frustrated” (40.4%) and “isolated” (33.1%). These results are consistent with previous studies that report that the lower one’s level of knowledge, the easier one may persuaded by irrational beliefs, conspiracy theories, or misinformation about quarantine and fall into negative emotions [35]. In addition, the stronger the negative emotions, such as anxiety, the more motivated the infection prevention behavior; however, such negative emotions can interfere with thinking processes that enable individuals to cope with the crisis, causing unscientific prevention behavior [35].

Therefore, for individuals who are not aware of their negative emotions, emotional cognition is an important cognitive process during crises, such as the pandemic, because it can prevent exaggerated preventive actions or unscientific quarantine.

#### Emotions related to diagnosis and treatment

Regarding the third area, the diagnosis and treatment of infectious diseases, similar emotions were shown in the two groups regardless of the level of understanding.

The highest percentage of emotions for the diagnosis and treatment of respiratory-related infectious diseases was "reliable” (43.3%). The linked emotions were "relieved” (32.7%) and "responsible" (29.9%).

Negative emotions, such as “complex” (16.9%), “sick” (13.0%), and “suspicious” (11.3%), were also linked. Interestingly, this result was the same across different levels of understanding of infectious diseases. These results may be due to the timing of the study—the early stages of the COVID-19 pandemic; specifically, the participants may have felt confident about diagnosis or treatment because the number of vaccines or and treatments for severe patients was on the rise, but they may also have felt confused about and questioned treatment, isolation, and diagnostic methods. However, compared to the initial stage, the prolonged COVID-19 situation is believed to have increased trust in medical staff and the government as the death rate and incidence rate have decreased and infections are now controlled. Indeed, confidence in medical staff and the government regarding diagnosis and treatment is crucial in a pandemic situation, as previous studies have shown that trust in government agencies has reduced COVID-19 mortality [36]. Notably, a study on Ebola also found that feelings of confidence in medical staff and the government were linked to embracing healthcare measures [37, 38].

This confidence is important because it leads to voluntary testing, reducing the spread of infectious diseases, and securing more treatment time. Therefore, the cultivation of a feeling of trust in the treatment of infectious diseases can improve the management of infectious diseases.

### Preventive practices based on understandings of infectious diseases

In this study, the practice rate of preventive actions according to the level of understanding of infectious diseases was analyzed, but no statistically significant difference was found. Although educational intervention is generally known to be effective in improving an individual’s knowledge, attitude, and behavior, its effect on information provision is uncertain due to the nature of unpredictable and threatening new situations, such as COVID 19. This result aligns with that of study that reported that while such a situation may involve some behavioral changes, it can be difficult to continuously practice practice preventive actions [1]. In addition, in the early stages of the COVID-19 pandemic, false information about infectious diseases spread rapidly around the world, and conspiracy theories related to the origin, prevention, diagnosis, and treatment of diseases appeared on social networking services (SNS), hindering the practice of correct preventive actions. These results are consistent with previous studies showing that uncertainty about the virus has hindered individual preventive behavior [35]. It is also in the same vein as the results of public health studies reporting that knowledge provision or education has increased knowledge recalls, but the increase in recalls does not necessarily lead to behavioral changes [39]. Scholars have also suggested that educational intervention affects behavioral changes when it is consistent with individual values or worldviews [40], when it appeals to altruistic motives [41], and when it is feasible and requires minor changes [42]. However, since COVID-19 prevention measures can be overwhelming and threatening and have involved restrictions on all aspects of daily life and social life, there may be a limit to this kind of action; for example, in the early stages of COVID-19, too much information can be confusing and overwhelming [43, 44].

Therefore, since our study did not find any difference in the practice of preventive actions depending on the level of understanding of infectious diseases, it suggests that information provision itself is more meaningful than the amount or level of information in the early stages of the pandemic. However, as the pandemic has continued for more than three years, and accurate information about the virus and preventive activities caused by vaccines are active, preventive actions are now considered to differ across levels of understanding, so further research is needed. Previous studies have also shown that the more knowledge an individual has about the characteristics, symptoms, and treatment of COVID-19, the less likely they are to have problems with their mental health [45]. Therefore, in the COVID-19 era, which has passed the initial stage, accurate knowledge and information delivery are required to enhance preventive actions, and continuous management and confirmation by the government and local communities are considered necessary.

## Conclusions

In summary, this study revealed that a relationship between emotion and understanding during the COVID-19 pandemic—individuals with high levels of COVID-19 related knowledge tended to feel general negative emotions, while individuals with low levels of COVID-19 related knowledge tended to feel fear or extreme fear; in addition, it also found that perceptions of prevention behavior differed depending on level of understanding.

This study’s findings are meaningful for managing mental and psychological health during the COVID-19 pandemic and also have a practical impact for the end of the pandemic. First, these findings suggest that effective communication strategies should be developed to check existing levels of understanding of diseases to improve personal quarantine during public health crises. Second, this study found that a lack of knowledge about COVID-19 reduces trust and yields negative emotions. In addition, it also found that people with negative emotions were more likely to demonstrate excessive or unscientific preventive actions. These findings suggest that it is necessary to decrease negative emotions, prevent unnecessary information, and alleviate fear.

There are some limitations to this study. First, the causal relationship between understanding and emotion is uncertain when cross-sectional information is used.

Second, to enhance emotional cognition, the study suggests including non-verbal elements, such as body gestures, paralanguage (e.g., tone of voice, accent), and physical changes (e.g., heartbeat, breathing, blushing). As emotional cognition can be expressed either verbally or non-verbally, further research should be done on non-verbal emotional cognition.

Third, the expressions of emotional cognition used in this study may differ across countries and cultures; further research needs to consider cultural contexts.

## Data Availability

The datasets analyzed during the current study are available from the corresponding author on reasonable request.
